# Behçet's Syndrome in a Chinese Pedigree of NLRP3-Associated Autoinflammatory Disease: A Coexistence or Novel Presentation?

**DOI:** 10.3389/fmed.2021.695197

**Published:** 2021-06-24

**Authors:** Jinjing Liu, Xin Yu, Chaoran Li, Yi Wang, Weihong Yu, Min Shen, Wenjie Zheng

**Affiliations:** ^1^Department of Rheumatology and Clinical Immunology, Chinese Academy of Medical Sciences and Peking Union Medical College, Beijing, China; ^2^National Clinical Research Center for Dermatologic and Immunologic Diseases, Ministry of Science & Technology, Beijing, China; ^3^State Key Laboratory of Complex Severe and Rare Diseases, Peking Union Medical College Hospital, Beijing, China; ^4^Key Laboratory of Rheumatology and Clinical Immunology, Ministry of Education, Beijing, China; ^5^Department of Rheumatology, Peking University Shougang Hospital, Beijing, China; ^6^Department of Otolaryngology, Peking Union Medical College Hospital, Beijing, China; ^7^Key Lab of Ocular Fundus Diseases, Department of Ophthalmology, Peking Union Medical College Hospital, Beijing, China

**Keywords:** *NLRP3*-associated autoinflammatory disease, Behçet's syndrome, vasculitis, pedigree analysis, whole-exome sequencing

## Abstract

**Objectives:** *NLRP3*-associated autoinflammatory disease (*NLRP3*-AID) and Behçet's syndrome (BS) both belong to autoinflammatory diseases and rarely co-occur. Here we reported a Chinese pedigree of *NLRP3*-AID presented with BS.

**Methods:** We recorded a Chinese pedigree of *NLRP3*-AID presented with BS. Whole-exome sequencing was performed to find the hereditary susceptibility gene, and Sanger sequencing was performed on a consecutive cohort of 30 BS patients. We also reviewed the English literature on vasculitis associated with *NLRP3*-AID.

**Results:** The proband was a 45-year-old Chinese Han woman. She and her 12-year-old daughter presented with recurrent fevers, cold-induced urticaria, oral, and genital ulcers, conjunctivitis, uveitis, optic atrophy, erythema nodosum, headache, and hearing loss. They were initially suspected of having BS, and both responded poorly to corticosteroids and immunosuppressants, while anti-TNF therapy was moderately effective. Pedigree analysis revealed another four relatives with similar symptoms, and a heterozygous *NLRP3* gene mutation c.1316C>T, p.Ala439Val was identified by whole-exome sequencing and Sanger sequencing. However, we did not discover *NLRP3* gene mutation by Sanger sequencing in a confirmative cohort of 30 BS cases. A few case reports of vasculitis coexisting with *NLRP3*-AID, including a case of glomerulonephritis, and five cases of retinal vasculitis, were summarized through literature review.

**Conclusions:** Our study is the first report of *NLRP3*-AID associated with BS. The coexistence of *NLRP3*-AID and BS reveals the extensive heterogeneity of the pathogenesis of systemic autoinflammatory diseases and calls for specific therapeutics.

## Introduction

The definition of systemic autoinflammatory diseases (SAIDs) is widening and now includes monogenic and polygenic disorders (1). *NLRP3*-associated autoinflammatory disease (*NLRP3*-AID) (formerly called cryopyrin-associated periodic syndrome, CAPS), an autosomal dominantly inherited SAID, encompasses a group of disorders with overlapping phenotypes including, in order of increasing severity, familial cold autoinflammatory syndrome (FCAS), Muckle-Wells syndrome (MWS), and chronic infantile neurological cutaneous articular (CINCA) syndrome. *NLRP3*-AID is caused by gain-of-function mutations of the NLR family pyrin domain containing-3 (*NLRP3*) gene on chromosome 1q44, which encodes a protein previously called cryopyrin, now known as the NOD-like receptor 3 (NLRP3) (2). The NLRP3 protein is mainly expressed in monocytes, macrophages, and neutrophils. It plays a vital role in regulating the native inflammatory response, as it represents a key component of the NLRP3-inflammasome complex, which is involved in pathogen-associated molecular patterns and danger-associated molecular patterns recognition and leads to IL-1β secretion and pyroptosis (3). *NLRP3*-AID is characterized by recurrent fever, urticarial rash, and non-infectious inflammation in the central nervous system, accompanied by arthritis/arthralgia, ocular inflammation, and sensorineural deafness. Vasculitis is a rare manifestation in patients with *NLRP3*-AID and is one of the challenging differential diagnoses of *NLRP3*-AID for they share common features such as intermittent fever, arthritis, cutaneous and ocular involvement.

Behçet's syndrome (BS) is a multifactorial inflammatory disorder characterized by recurrent attacks affecting the mucocutaneous tissues, eyes, joints, blood vessels, brain, and gastrointestinal tract. It is classified as variable vasculitis involving both arteries and veins of all sizes. Nowadays, BS is widely accepted as a polygenic SAID since it has several autoinflammatory features, with a robust innate immune activation and overexpression of pro-inflammatory markers (4–6).

*NLRP3*-AID can hardly be seen with vasculitis, especially BS, though both belong to SAIDs. Herein, we reported a Chinese pedigree with typical manifestations of *NLRP3*-AID who uniquely had BS. We also conducted a systemic review of *NLRP3*-AID with vasculitis.

## Patients and Methods

The proband was referred to our tertiary medical center, and the complete medical records were documented, including the pedigree and the disease histories of kindred. To find the hereditary susceptibility gene, whole-exome sequencing by next-generation sequencing (NGS) was performed on blood samples from the proband and family members. The result was analyzed by Clinical Sequence Analyzer (Genuity Science) by filtering the Minor Allele Frequency (MAF), and the filtered gene was categorified based on Human Gene Mutation Database (HGMD). We also performed Sanger sequencing on the proband, the proband's family members, and a consecutive cohort of 30 BS patients to confirm NGS findings, the result of which was demonstrated by the software DNAMAN. The study was performed in Chigene (Beijing) Translational Medical Research Center and approved by the Institutional Review Board of Peking Union Medical College Hospital and performed according to the Declaration of Helsinki. Informed consent was obtained from the participants.

**Figure 1:**
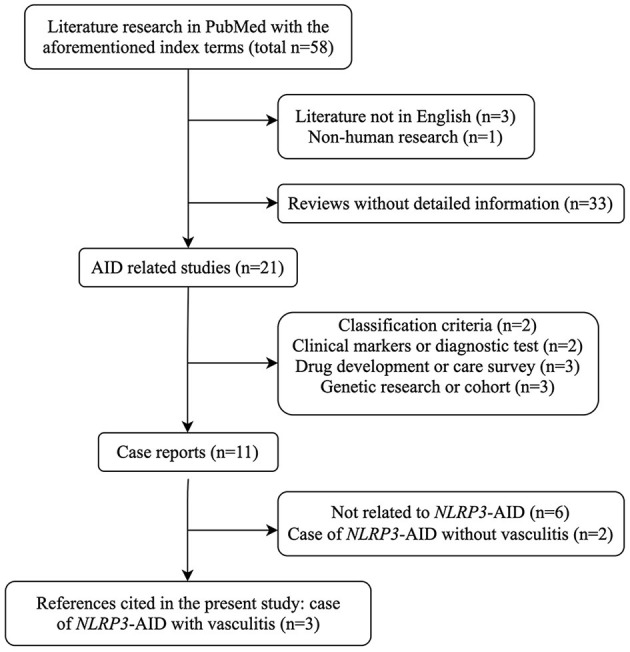
Flow chart of literature research strategy with the index terms mentioned. AID, autoinflammatory disease; *NLRP3*-AID, *NLRP3*-associated autoinflammatory disease.

We conducted a systematic English literature search in Pubmed NCBI, using the index terms “*NLRP3*-associated autoinflammatory disease AND vasculitis,” OR “*NLRP3*-associated autoinflammatory disease AND Behçet's syndrome,” OR “cryopyrin-associated periodic syndrome AND vasculitis,” OR “cryopyrin-associated periodic syndrome AND Behçet's syndrome,” OR “MWS AND vasculitis,” OR “CINCA AND vasculitis” (Figure 1). There were only three relevant case reports on vasculitis associated with *NLRP3*-AID.

## Results

### Case Description

The proband was a 45-year-old Chinese Han woman. She developed cold-induced urticaria at the age of six, accompanied by recurrent chills and high fevers exacerbated at winter nights. Each episode lasted several hours to days, with intervals of several days to weeks. She also had intermittent poly-arthralgia and arthritis involving bilateral knee, ankle, and elbow joints. She presented hearing loss, sinusitis, and otitis with perforated tympanic membranes. Bilateral sensorineural hearing loss, left conductive hearing loss, and vestibular dysfunction associated with otitis were diagnosed by an otolaryngologist. At 37 years of age, she suffered from recurrent painful oral and genital ulcers, resulting in gingival recession and teeth loss. At the age of 40, erythema nodosa on lower extremities was noted and confirmed pathologically, and anterior uveitis was diagnosed shortly after. Gradually, she complained of headache, dizziness, numbness of extremities, myalgia, and fatigue. Brain magnetic resonance imaging (MRI) showed bilateral subcortical lacunar infarctions in the frontal and parietal lobes. Lumbar puncture revealed an intracranial pressure of 135 mmH_2_O. The routine and biochemistry testing of cerebrospinal fluid was normal and pathogenic examination was negative. Electroencephalogram revealed epileptiform discharges and electromyogram found small fiber sensory neuropathy. She also had recurring abdominal pain restricted to the right lower quadrant. Colonoscopy identified multiple hyperplastic bulges in the colon, and biopsy proved numerous foamy cell aggregations in the lamina propria and focal lymphoid tissue hyperplasia. On physical examination, she had mild frontal bossing and conjunctival congestion. Mucosal ulceration and missing teeth were noted. She had an unsteady gait for her dizziness. BS was suspected according to the 2014 International Criteria for Behçet's Disease (ICBD) (7), so glucocorticoids and immunosuppressants were given sequentially, including methotrexate, thalidomide, mycophenolate mofetil, hydroxychloroquine, cyclophosphamide, cyclosporine, azathioprine, and colchicine, but with limited efficacy. Mucosal ulcers and arthritis waxed and waned with fluctuating inflammatory markers such as erythrocyte sedimentation rate (ESR) and C-reactive protein (CRP).

Complete blood count (CBC), urine analysis, and complete biochemistry panel were all within normal ranges. Serological autoantibodies including anti-nuclear antibody, anti-extractable nuclear antibodies, antineutrophil cytoplasmic antibodies (ANCA), rheumatoid factor, anti-cyclic citrullinated peptide antibodies were all negative. ESR was normal, while CRP was increased to 39.7 mg/L (normal range 0–3). Bilateral sensorineural hearing loss was proved by pure tone audiometry. Funduscopic examination was non-specific.

**Figure 2:**
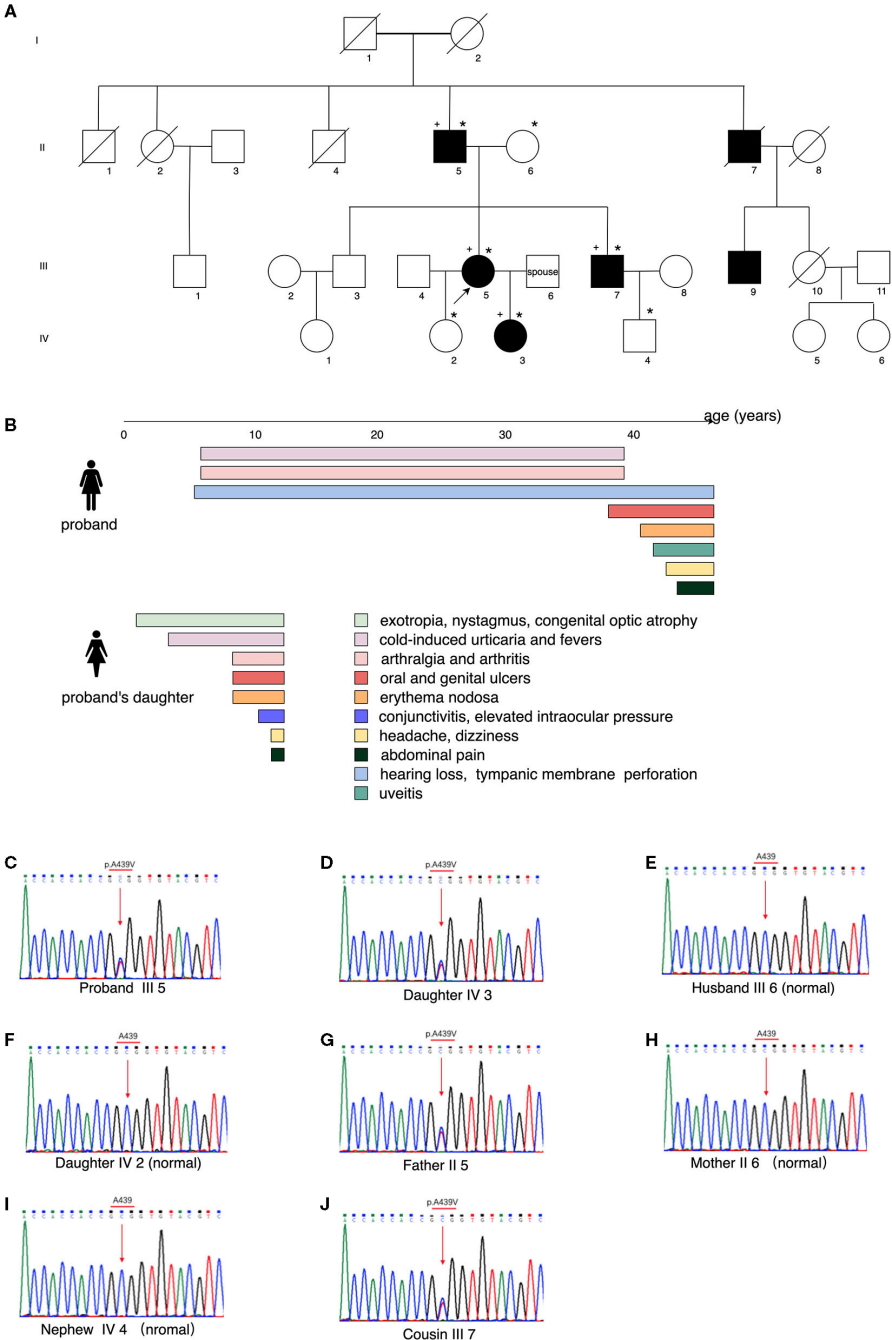
Pedigree analysis, clinical manifestations of the proband and her daughter, and the results of whole-exome sequencing. **(A)** Genealogical tree of the family, showing the affected individuals (filled symbols), healthy subjects (empty symbols), the proband (III 5, black arrow), DNA samples collected (*marked, to which individual III 9 refused), subjects with positive *NLRP3* gene mutation (+ marked). **(B)** Manifestations of the proband and her daughter, with colored bands representing the duration of symptoms. **(C–J)** Whole-exome sequencing by Next Generation Sequencing of the *NLRP3*-AID patients, showing the heterozygous mutation *NLRP3* (NM_001243133.1): c.1316C>T (p.Ala439Val) and that of the controls.

Pedigree analysis revealed five relatives who had similar symptoms, including her father (II 5), an uncle (II 7), a brother (III 7), a cousin (III 9), and her daughter (IV 3) (Figure 2A). They all had recurrent fevers and cold-induced urticaria. Her father and her brother also had polyarthralgia. Her uncle had oral ulcers and died of nasopharyngeal carcinoma. Her daughter presented with a more severe form of manifestations, such as cold-induced urticaria with chills and high fever, conjunctivitis, congenital optic atrophy, mucosal ulcers (oral, genital, and perianal), erythema nodosa and folliculitis. She also suffered from bilateral knee arthritis and intermittent abdominal pain. At 11 years old, she had two episodes of acute headache with nausea, vomiting, and blurred vision. Brain MRI showed a high-intensity signal on the hippocampus, and lumbar puncture revealed normal results of intracranial pressure and cerebrospinal fluid, while nocturnal epileptiform discharge was noticed on the electroencephalogram. Elevated intraocular pressure was confirmed by the ophthalmologist. The results of pure tone audiometry were normal. Laboratory tests found fluctuating inflammatory markers, intermittent urine erythrocyte and protein. Serological autoantibodies including ANCA were all negative. Levels of immunoglobulins and complements were within the normal range. The disease course and manifestations of the proband and her daughter were indicated in Figure 2B.

DNA samples were collected from the alive affected individuals of the family and the proband's spouse, except for individual III 9, who refused to test. Whole-exome sequencing by NGS identified a heterozygous mutation c.1316C>T, p.Ala439Val of the *NLRP3* gene (NM_001243133.1) in the proband (III 5), her father (II 5), her brother (III 7), and her daughter (IV 3) (Figures 2C–J). Combined with the clinical manifestations and genetical finding, the proband and her daughter were diagnosed as a moderate type of *NLRP3*-AID (MWS) (8, 9).

The proband and her daughter both received TNF-α inhibitors (subcutaneous etanercept 50 or 25 mg weekly, separately), azathioprine, and low-dose steroids. After 6-months of therapy, her fevers, skin lesions, oral and genital ulcers, arthritis, headache, and dizziness alleviated, no uveitis occurred, CRP decreased to the normal level. However, her hearing loss remained, and she still had occasional conjunctivitis, arthralgia, and myalgia. Her daughter's dermatitis, mucosal ulcers, arthritis, and headaches had also improved, and ESR and CRP levels were normal.

### Screening of Mutation in a Cohort of BS Patients

Then we screened the mutation mentioned above by Sanger sequencing in a consecutive cohort of 30 treatment-naïve or active BS cases that fulfilled the 2014 ICBD criteria, which did not reveal *NLRP3* p.Ala439Val mutation. The cohort contained 20 men and 10 women, with a mean age of 39 ± 12 years. Oral ulceration was presented in all patients, followed by skin lesions (*n* = 13, 43.3%, erythema nodosa or folliculitis), vascular/cardiac involvement (*n* = 13, 43.3%), gastrointestinal ulcers (*n* = 10, 33.3%), genital ulcers (*n* = 8, 26.7%). Additionally, neurological involvement, uveitis, and epididymitis were observed in 3 (10%), 3 (10%), and 1 (3.3%), respectively.

### Literature Review

Case reports revealed rare manifestations of vasculitis coexisting with *NLRP3*-AID (Table 1). A German girl with CINCA developed glomerulonephritis and vasculitis other than amyloidosis at age 12. Her renal function was stabilized with cyclophosphamide pulse therapy followed by corticosteroids and multiple immunosuppressants, while her meningitis, uveitis, and CRP levels significantly improved with IL-1-receptor antagonist anakinra, other than TNF-α monoclonal antibody therapy (10). Two girls from India (11) and Argentina (12) with CINCA suffered from retinal vasculitis and were successfully controlled with glucocorticoids and azathioprine. In a report focusing on ocular manifestations of CINCA, three out of 31 patients developed retinal vasculitis (13).

**Table 1:** Vasculitis associated with *NLRP3*-AID and comparison of *NLRP3*-AID with Behçet's syndrome.

**Characteristics**	**Behçet's Syndrome**	***NLRP3*-AID**	**Case 1 (the proband)**	**Case 2 (the proband's daughter)**	**Ebrahimi-Fakhari (10)**	**Russo (12)**	**Khemani (11)**
Diagnosis			MWS	MWS	CINCA	CINCA	CINCA
*NLRP3* mutation	–	+	A439P	A439P	A439V	N/A	F309S
Gender	M:F = 1.4:1 (28)	M:F = 5:2 (25)	Female	Female	Female	Female	Female
Nationality	Affects races along the “silk road”	Affects all races, many are of European decent	Chinese	Chinese	German	Argentinean	Indian
Age (years)			45	12	23	3.5	7
Age at onset of disease	Young and middle-aged	Neonatal/infancy	6 years	6 months	Within the first week after birth	Within the first day of life	The second day of life
Recurrent oral ulcers	+	Occasionally	+	+	–	–	–
Skin lesions	Erythema nodosum and pathergy	Cold-induced urticarial rash	Cold-induced urticarial rash, erythema nodosum	Cold-induced urticarial rash, erythema nodosum, perianal ulcers, folliculitis	Cold-induced urticarial rash	Cold-induced urticarial rash	Cold-induced urticarial rash
Genital ulcers	+	–	+	+	–	–	–
Ocular lesions	Typical uveitis	Papilledema, uveitis, iritis, typically conjunctivitis	Uveitis, conjunctivitis	Conjunctivitis, elevated intraocular pressure, and blurred vision	Uveitis, optic neuritis, optic nerve atrophy	Retinal vasculitis, papilloedema	Retinal vasculitis
Arthritis	+	+	+	+	+	+	+
Gastrointestinal involvement	Intestinal ulcers	Abdominal pain	Right lower quadrant pain	Abdominal pain	–	Jaundice, hepatomegaly	–
Vascular lesions	Variable, some with cardiac involvement	Rarely pericardial effusions	–	–	–	–	–
Nervous system lesions	Parenchymal and non-parenchymal lesions	Headaches, chronic aseptic meningitis, high intracranial pressure, papilledema	Headache, dizziness, lacunar infarctions, small fiber sensory neuropathy	Binocular exotropia, nystagmus, congenital optic atrophy, acute headache, hippocampus high-intensity signal on MRI	Chronic aseptic meningitis, diffuse cortical atrophy, and ventriculomegaly on MRI	Mild hydrocephalous and cortical atrophy on CT scan	Cerebellar atrophy on MRI
Recurrent fevers	Irregular	Regular or with distinct triggers	+	+	+	+	+
Sensorineural hearing loss	–	+	+	+	+	–	–
Renal involvement	Rare, amyloidosis	Amyloidosis	–	Proteinuria	Pauci-immune crescent glomerulonephritis with diffuse extracapillary necrosis and vasculitis without amyloidosis	–	–

*CINCA, chronic infantile neurological cutaneous articular syndrome; F, female; M, male; MWS, Muckle-Wells syndrome, NLRP3, NLR family pyrin domain containing-3 gene; NLRP3-AID, NLRP3-associated autoinflammatory disease*.

## Discussion

Whole-genome sequencing and Sanger sequencing revealed that the proband and her three relatives had a heterozygous *NLRP3* A439V mutation, which was among the first identified mutations in the gene *NLRP3*, and the others include V198M, E627G, and A352V (14). The gain-of-function effect of the A439V mutation led to high levels of IL-1β secretion, which was proved to be pathogenic of *NLRP3*-AID (15). In combination with the proband's clinical presentations, including periodic fever after exposure to cold, urticaria, arthritis, headache, and hearing loss, the diagnosis of *NLRP3*-AID was made. Due to the absence of IL-1 antagonists in China, TNF-α inhibitors were given and showed moderate effects.

Intriguingly, the proband and her affected relatives in our study had simultaneous BS-like manifestations. Recurrent aphthous ulcers, perianal ulcers, folliculitis, erythema nodosa, and uveitis could meet the diagnostic criteria for BS. Given that no biomarker could be diagnostic for BS, several sets of diagnostic criteria have been published and evaluated in clinical practice, and the diagnosis sometimes needs thorough differential diagnosis and exclusion of mimicking conditions. The 2014 ICBD criteria are the most recent, in which ocular lesions, oral and genital aphthosis are each assigned 2 points, a patient scoring 4 points or above can be classified as having BS. According to the international team for the revision of ICBD, the new criteria showed satisfactory sensitivity (93.9%) and acceptable specificity (89.6%) in the training dataset (7). BS is widely accepted as a syndrome rather than a certain disease, for its unknown etiologies and various phenotypes, and predominant temporal and spatial heterogeneity. Although mucocutaneous involvement is a hallmark of BS, it is reasonable that our two patients presented as BS-like syndrome during their disease course, yet they eventually developed the complete spectrum of *NLRP3*-AID. Indeed, there were some similar characteristics of *NLRP3*-AID and BS, such as recurrent fever, arthritis, and aseptic meningitis leading to headache and dizziness. Due to the accumulating evidence with IL-1 targeted therapies in BS, it is regarded as a polygenic SAID (16). The distinct manifestations of BS and *NLRP3*-AID were reviewed in Table 1.

The consensus was established that phenotype-genotype associations composed distinctive features of SAIDs, thus reflected in the classification criteria (17). Approximately 100 pathogenic *NLRP3* mutations have been reported in *NLRP3*-AID patients with solid genotype–phenotype correlation along the disease continuum from mild type (FCAS), moderate type (MWS) to severe type (CINCA) (8). In addition, some special *NLRP3* variants may have an association with clinical phenotype. For example, according to a recent report from our center, *NLRP3* T348M was related to severe neurological involvements as sensorineural hearing loss, chronic aseptic meningitis, hydrocephalus, and brain atrophy (18). A439V reported a fairly consistent genotype–phenotype correlation with FCAS/MWS (8). Specifically, urticaria-like rash, and ocular inflammation as conjunctivitis and uveitis, were the phenotypes positively correlated with A439V mutation in a large German family with FCAS/MWS-overlap syndrome (19), which was consistent with the manifestations of our patients.

Although certain mutations in *NLRP3* may contribute to the pro-inflammatory cytokine profiles as which has been reported in Turkish and Italian BS patients (20, 21), the clinical characteristics of the pedigree in our study could not fit typical BS phenotypes. First, two individuals from this family presented with BS, but genetic testing did not identify gene mutations that were associated with BS susceptibility, e.g., histocompatibility leukocyte antigen (HLA)-B^*^51, IL23R-IL12RB2 and IL10, Toll-like receptor (TLR) 2 and TLR4, and *TNFAIP3* gene polymorphisms (22). Instead, *NLRP3* gene mutation found in the affected family members was not reported to increase the genetic risk of BS. Nevertheless, our expanded screen in a cohort of 30 cases of BS did not reveal *NLRP3* mutations. Second, the patients in our study had earlier ages of disease onset. The reported mean age of BS onset ranges from young to middle-aged years, whereas the proband and her daughter developed typical symptoms at 6 years and 5 months, respectively. Third, since their BS-like manifestations were limited to mucosal ulcers, erythema nodosa, and folliculitis, the evidence for “BS” diagnosis was weak with mild uveitis, meningitis, and absence of vascular or gastrointestinal manifestations. Fourth, they had a moderate response to TNF-α antagonists, yet not to the combination therapy of glucocorticoids and immunosuppressants. This could probably be attributed to the coexisting *NLRP3*-AID.

Vasculitis is one of the major manifestations of BS, but not *NLRP3*-AID. Through literature review, SAIDs with vasculitis were seen in case reports, but the causes of vasculitis associated with monogenic SAIDs are still debated. On the one side, certain monogenic SAIDs mimic vasculitis. For instance, the newly described loss-of-function mutation in *TNFAIP3* leads to a BS-like disease, haploinsufficiency of A20 (HA20) (23); deficiency of adenosine deaminase 2 (DADA2) usually presents with polyarteritis nodosa vasculopathy (24). On the other side, vasculitis accompanying monogenic SAIDs have recently been reported in familial Mediterranean fever (FMF), tumor necrosis factor receptor-associated periodic fever syndrome (TRAPS), and STING-associated vasculopathy with onset in infancy (SAVI) (6). However, case reports revealing vasculitis mainly focused on retinal vasculitis and glomerulonephritis. In our cohort of adult *NLRP3*-AID, we have reported that only 29% of patients had occasional oral aphthous ulcers, but no genital or perianal ulcers, folliculitis, or uveitis (25). The same *NLRP3* A439V mutation was found in 15 of 37 German family members (41%), and they all presented with the typical clinical features for *NLRP3*-AID (19), but not BS-like symptoms. Therefore, we think BS is coexisting with *NLRP3*-AID in the pedigree patients in our study, rather than being regarded as an integral part of *NLRP3*-AID.

Since both *NLRP3-*AID and BS are corticosteroid-dependent autoinflammatory conditions, biological agents have recently been widely used in these disorders. Treatment of *NLRP3-*AID targets on inhibition of the inflammasome-derived cytokine IL-1β (9), which drives autoinflammatory processes and also acts on the effector cells of the adaptive immune system, while TNF-α inhibitors are widely accepted as efficacious therapy for refractory BS. Meanwhile, TNF-α inhibitors are second-line options for other monogenic autoinflammatory diseases such as TRAPS, MKD, and FMF (26), therefore, they may be an alternative approach to *NLRP3*-AID. Previous studies have revealed that TNF-α could promote the *NLRP3* inflammasome activation through the NF-κB pathway, leading to caspase-1 activation and IL-1β secretion (27). Additionally, IL-1 inhibitors are not commercially available in China. Thus, we tried etanercept for the proband and her daughter, and they both had a moderate response. Nowadays, novel therapeutic targeting NLRP3 is rapidly progressing (16), bringing new hope for SAIDs patients.

The limitation of our study was that the sample size of our BS cohort for the *NLRP3* gene evaluation was relatively small. Although it was a consecutive cohort, the patients selected mainly had mucocutaneous, vascular, and gastrointestinal involvement. Phenotypes of uveitis and central nervous system involvement were lacking, whereas these two organ involvements were unique features of *NLRP3*-AID, especially MWS. Further evaluation should include adequate patients, and sufficient BS phenotypes to eliminate selection bias.

In conclusion, this is the first report of *NLRP3*-AID associated with BS in a Chinese family, including several affected members, all harboring the A439V mutation in the *NLRP3* gene. Further studies about the pathophysiology of BS and monogenetic SAIDs are needed for better recognition of the connection between these two conditions.

## Data Availability Statement

The original contributions presented in the study are included in the article/supplementary material, further inquiries can be directed to the corresponding author/s.

## Ethics Statement

The studies involving human participants were reviewed and approved by the institutional ethics review board of Peking Union Medical College Hospital (S-443). Written informed consent to participate in this study was provided by the participants' legal guardian/next of kin.

## Author Contributions

WZ and MS followed up the patients, designed the study, and revised the manuscript. JL also followed up the patients and drafted the manuscript. XY and CL performed the experiments, sample collection, and data analysis. YW and WY provided professional disease assessment. All authors have read and approved the final manuscript.

## Conflict of Interest

The authors declare that the research was conducted in the absence of any commercial or financial relationships that could be construed as a potential conflict of interest.
